# A community case study of pedestrian environment features of neighborhood walkability and built environment-related 311 service requests surrounding public housing developments serving low-income Boston residents

**DOI:** 10.3389/fpubh.2026.1805971

**Published:** 2026-05-12

**Authors:** Paige Krasnoff, Lisa M. Quintiliani, Siyao Du, Brennan Rhodes-Bratton, Jennifer Murillo, Iolando Spinola, John Kane, Jemar R. Bather, Melody S. Goodman, Julien Dedier

**Affiliations:** 1 School of Medicine, Tufts Medical Center, Tufts University, Boston, MA, United States; 2 New York University Steinhardt School of Culture, Education, and Human Development, New York, NY, United States; 3 New York University School of Global Public Health, New York, NY, United States; 4 WalkMassachusetts, Boston, MA, United States; 5 Boston Housing Authority, Boston, MA, United States; 6 Boston University Chobanian and Avedisian School of Medicine, Boston Medical Center, Boston, MA, United States

**Keywords:** built environment, neighborhood walkability, physical activity, public housing, urban

## Abstract

Physical activity is a modifiable health behavior influenced by neighborhood walkability. In-person field audits of pedestrian environment features of neighborhood walkability were conducted at the start of an ongoing multilevel, multicomponent physical activity promotion intervention called Community Walks, taking place in 12 low-income public housing developments (PHDs) in Boston, MA, USA. Following these audits, we then explored alternative, remote methods of walkability assessment. One option is the publicly available database of service requests submitted by individuals in municipalities across the U.S. by calling ‘311’ to report problems that can present obstacles to physical activity (e.g., potholes, broken crosswalk signals). In this Community Case Study, we examined the cross-sectional relationship between pedestrian environment features and neighborhood walkability scores in the 12 low-income PHDs in Community Walks, and the frequency of 311 service requests within a 0.1-mile radius of each PHD’s address. In adjusted analyses, a one-unit increase in the overall walkability score was associated with a 12% (95% CI: 1.03–1.22) increase in the frequency of 311 service requests. We then describe possible explanations of these findings and implications for community-based physical activity interventions. For example, pedestrian advocacy training is an intervention component that could raise residents’ awareness of the 311 system and encourage its use to increase reporting of walkability concerns. This study represents an exploratory starting point for future multi-level physical activity interventions in which 311 data could inform intervention programming aimed at increasing health-promoting physical activity behaviors.

## 1 Introduction

Physical activity is a modifiable behavior with well-established benefits for reducing chronic disease risk (1). Studies consistently show that community physical activity levels increase in walkable neighborhoods (2), yet many communities remain poorly designed to support sustained activity levels (3). Acquiring accurate data on the built environment can be labor-intensive and costly, especially at the level of detailed neighborhood features (4–7). Developing large-scale, cost-effective assessments to measure these environmental features remains a major challenge for public health research and could improve how policymakers, public health practitioners, community residents, and other interested parties advocate for and direct resources toward infrastructure improvements that support physical activity.

‘Community Walks’ is a trial employing a cluster randomized design to evaluate a multilevel, multicomponent intervention program to increase moderate-intensity physical activity among individuals living in low-income public housing developments (8). Residents of urban public housing developments in the U.S. are disproportionately affected by physical activity-responsive chronic health conditions such as obesity, cardiovascular disease, asthma, diabetes, and mental illness (9, 10). Previous research has found that ‘macroscale’ and ‘microscale’ characteristics of the built environment influence health outcomes, Cain et al. (4) and levels of physical activity tend to be higher in public housing developments (PHDs) with fewer structural barriers to exercise (10–12). Macroscale characteristics are the large-scale structure and organization of cities and regions, concerning how land is used and spaces are interconnected, such as zoning policies (13). Microscale features are detailed attributes of the pedestrian environment that directly affect human interaction and behavior on a personal level, such as the condition of sidewalks, street lighting, wayfinding signage, and landscaping (13). In urban settings, microscale features have a significant influence on physical activity (14, 15).

Features of the microscale environment are most commonly assessed through field audits, which are regarded as the gold standard for evaluating neighborhood pedestrian environments (16). Field audits are structured, on-site evaluations performed by trained observers who systematically rate relevant microscale characteristics. Incorporating standardized checklists, such as the Microscale Audit of Pedestrian Streetscapes (MAPS), Sallis (17) strengthens validity and enables comparison across diverse geographic locations. Field audits using MAPS were conducted to characterize the pedestrian environment features of the walkability of neighborhoods included in the Community Walks intervention.

After field audits for the Community Walks program were complete, the study team decided to explore remote alternatives to characterizing neighborhood walkability because field audits are both labor- and time-intensive processes. These alternatives include panoramic Global Positioning System-based (GPS) applications such as Google Street View™, (which have increased convenience by allowing online environmental assessments, but still require manual evaluation and data analysis (5)) and artificial intelligence technologies (which can automatically analyze streetscape images but are costly and have variable accuracy (6, 7)). A third potential source of information on the built environment is the ‘311’ system used in many U.S. municipalities (18). This system offers a simple, convenient method for the public to submit non-emergency service requests, such as potholes, broken streetlights, or uncollected trash, to their local government. It also enables municipalities to efficiently catalogue, process, and address these requests. Originally accessible by dialing ‘311’ on a telephone, the system can now be reached by phone, text messaging, social media, internet, or through a mobile phone application (12). Each request is categorized systematically, 311 Service Requests - CRM Value Codex (19) which helps local governments track trends over time, and has garnered interest from the research community. For example, 311 data have been used in algorithms to identify urban blight (20), analyze variations in housing prices (21), and serve as predictors of neighborhood distress and substance abuse (22). Three hundred and eleven data may provide publicly available, low-cost, and accurate data sources to capture microscale attributes of neighborhood walkability that inform community health program implementation, advocacy, and resource allocation.

Despite the potential of municipal 311 service request systems to capture resident-reported issues related to the built environment, these data sources have rarely been leveraged in research on physical activity or health equity. The lack of studies connecting 311 data with pedestrian environment features of neighborhood walkability, especially within public housing communities, is a notable gap in the literature. Therefore, we examined the relationship between 311 service requests related to pedestrian environment features (microscale attributes) of neighborhood walkability at baseline among the 12 PHDs participating in the Community Walks program in Boston, MA, USA. We also discuss how findings could inform the implementation of Community Walks and future multi-level physical activity community programs.

## 2 Context

### 2.1 Context design and setting

Community Walks is an ongoing community-based trial that employs a cluster-randomized design to evaluate a multilevel, multicomponent intervention to increase moderate-intensity physical activity among individuals with low income residing in Boston’s PHDs (8). There are 12 PHDs in Community Walks, divided into four intervention groups: environmental intervention alone (3 developments), individual intervention alone (3 developments), environmental plus individual intervention (3 developments), and an assessment-only control group (3 developments). The environmental intervention includes community health worker (CHW)-led activities, pedestrian advocacy training sessions, and maps/wayfinding signage displaying walking routes. The individual intervention is a 12-week automated telephone program to increase motivation and self-efficacy. Intervention activities are designed to be enjoyable, safe, and accessible and are promoted through collaboration with front-line housing staff. The current study analyzed the geographic area within a 0.1-mile radius of each PHD’s address between January 31, 2022, and January 31, 2023. This radius was selected to be consistent with other neighborhood spatial research (23). All study procedures were approved by the Institutional Review Board of Tufts Medical Center. This report conforms to STROBE (Strengthening the Reporting of Observational Studies in Epidemiology) guidelines (24).

### 2.2 Data collection, processing, and analysis

#### 2.2.1 Dependent variable: assessment of 311 service requests

The City of Boston’s 311 service connects constituents (e.g., residents and visitors) with the local government for non-emergency help and information. Service requests are submitted by calling 3–1-1 or a local phone number, using the BOS: 311 app to upload photos, or by filing an online request (25). During the submission, the constituent selects from nine service categories (i.e., litter and trash, health hazards, street and park damage, vehicle/parking, lights, illegal graffiti, trees, flooding and drainage, and general) to file the request. Three hundred and eleven requests from a year long period (1/31/2022–01/31/2023) were obtained. Each service request is categorized under one of 59 possible ‘reason’ variables within the 311 database (e.g., street cleaning, streetlights, signs, and signals) (19). When restricting the 311 dataset to the selected timeframe and geographic boundaries, service requests fell into 34 of these reason categories. The categories were then compared to the Ecological Model of Four Domains of Active Living (11) to identify service requests associated with both physical activity and the built environment. Using a consensus method, authors LQ, JD, and PK identified reasons that corresponded to domains in this model (see Table 1). Discrepancies were discussed until reaching unanimous agreement. The results below are presented separately for all service requests and, in a sensitivity analysis, for those corresponding to an Ecological Model domain.

**Table 1:** City of Boston’s Department of Boston 311 incident reasons, with indication of inclusion in domain analysis, and corresponding ecological model domain.

Reasons	In domain analysis	Ecological model domain	
Abandoned bicycle	**✓**	Behavior settings: access and characteristics	Active transport: neighborhood
Administrative and general requests		n/a	
Animal issues	**✓**	Perceived environment: behavior settings: access and characteristics	Safety active recreation: Neighborhood
Boston bikes	**✓**	Behavior settings: access and characteristics	Active recreation: neighborhood
Building		n/a	
Catchbasin		n/a	
Code enforcement	**✓**	Policy environment	
Employee and general comments		n/a	
Enforcement and abandoned vehicles	**✓**	Behavior settings: access and characteristics	Active recreation: neighborhood
Environmental services	**✓**	Perceived environment	Attractiveness and safety
Fire hydrant		n/a	
Generic noise disturbance	**✓**	Perceived environment	Safety, Comfort, Perceived crime
Graffiti	**✓**	Perceived environment behavior settings: access and characteristics	Attractiveness and safety Active recreation: neighborhood
Health		n/a	
Highway maintenance		n/a	
Housing	**✓**	Perceived environment	Safety, comfort, convenience
Needle program	**✓**	Perceived environment Behavior settings: access and characteristics	Comfort, perceived crime Active recreation: neighborhood
Noise disturbance	**✓**	Perceived environment	Safety, comfort, perceived crime
Notification		n/a	
Operations		n/a	
Park maintenance and safety	**✓**	Policy environment Behavior settings: access and characteristics Perceived environment	Public recreation investments and park policies Active recreation: neighborhood Safety
Parking complaints	**✓**	Behavior settings: access and characteristics	Active transport: Neighborhood
Pothole	**✓**	Behavior settings: access and characteristics	Active recreation: Neighborhood
Recycling		n/a	
Sanitation	**✓**	Behavior settings: access and characteristics	Active recreation and Active transport: neighborhood
Sidewalk cover/manhole	**✓**	Behavior settings: access and characteristics	Active transport: neighborhood
Signs and signals	**✓**	Behavior settings: access and characteristics	Active transport: information during transport
Street cleaning	**✓**	Behavior settings: access and characteristics	Active transport: neighborhood
Street lights	**✓**	Behavior settings: access and characteristics	Active recreation: neighborhood and active transport: information during transport
Traffic management and engineering	**✓**	Policy environment Behavior settings: access and characteristics	Traffic demand management, transport investments and regulations Active transport: Information during transport
Trees	**✓**	Behavior settings: access and characteristics perceived environment	Active recreation: recreation environment attractiveness and comfort

#### 2.2.2 Independent variable: pedestrian environment features of neighborhood walkability

Between June 2022 and February 2024, trained personnel at WalkMassachusetts, a non-profit organization committed to improving local pedestrian infrastructure, evaluated walkability in the 12 PHDs using the Microscale Audit for Pedestrian Streetscapes-Abbreviated (MAPS-A) (17). This tool consists of structured questions addressing elements such as sidewalk presence, width, continuity, maintenance, and streetscape features (26). The assessments involved completing the MAPS-A survey while walking along three routes of varying distances per PHD. When selecting routes to evaluate, WalkMassachusetts staff considered key destinations within and around the developments (e.g., grocery stores), as well as pedestrian-friendly amenities such as shade, benches, and sidewalk conditions to ensure the routes were both practical and safe and could accommodate a wide range of participants of varying abilities and age groups. Data collected during the evaluations were entered into REDCap on iPads in real time.

We calculated five total positive subscales according to the MAPS-A manual: destinations and land use, streetscape, segments, aesthetics and social attributes, and crossings (Table 2). For the total positive destinations and land use subscale (range: 0–33), higher scores indicated greater land use diversity and access to destinations. For total positive streetscape (range: 0–9), higher scores indicated better quality street infrastructure. For total positive segments (range: 0–23), higher scores indicated greater environmental and streetscape quality of that segment. For positive aesthetics and social attributes (range: 0–3), higher values indicated higher quality of street aesthetics and sociability. For total positive crossings (range: 0–11), higher scores indicated improved crossing quality and accessibility. For routes with multiple streetscapes, segments, and crossings, mean scores were calculated and used in the analysis (17, 27).

**Table 2:** Components of MAPS-A subscale scores and overall scores used in analysis.

Positive destinations and land use	Total positive streetscape	Total positive segments	Positive aesthetics/ social attributes	Total positive crossings
Residential mix	Traffic calming (signs, circles, speed tables, speed humps, curbs)	Positive building height and setbacks	Hardscape	Crosswalk amenities (crossing aids, marked crosswalk, curb extensions, high visibility striping, different material)
Shops	Street amenities (overhangs, trash bins, place to sits, bike tracks)	Sidewalks	Softscape	Intersection control/signage (traffic circle, pedestrian walk signals, push buttons, countdown signal)
Restaurant-entertainment	Streetlights	Buffers	Landscaping maintenance	Curb quality/presence (pre-and post-crossing curbs)
Institutional-Service	Driveways/Alleys	Bike infrastructures		
Worship land uses	Transit tally (benches, shelters)	Trees		
School land uses		Informal path or shortcut positive		
Public recreation		Building-height to road-width ratio		
Positive destinations and land use				

Overall microscale positive	Overall total score	Overall microscale positive (active transport)	Overall total score (active transport)
Total positive streetscape	Positive destinations and land use	Total positive streetscape	Positive destinations and land use
Total positive segments	Total positive streetscape	Total positive segments	Total positive streetscape
Positive aesthetics/social	Total positive segments	Total positive crossings	Total positive segments
	Positive aesthetics/social		Total positive crossings

We also derived four overall summary scores based on prior literature (16). Higher values on the overall microscale positive score (range: 0–35) indicated greater environmental and neighborhood appeal. Higher values on overall microscale positive for active transport (range: 0–43) indicated better infrastructure and environment for active transportation. Higher values on the overall total score for active transport (range: 0–76) indicated an environment more conducive to active transportation, while higher values on the overall total score (range: 0–68) indicated a generally better pedestrian environment.

#### 2.2.3 Covariate

The number of units in a given PHD was considered a marker of PHD size, which was expected to influence the frequency of 311 requests. PHD unit counts were obtained from each PHD’s website.

### 2.3 Statistical analysis

Data analysis was conducted using R version 4.4.1. For each PHD, we calculated positive MAPS-A subscale scores and overall scores, as well as the number of 311 service requests. Because of their overlap, the total positive destinations and land use subscale scores and the four overall summary scores were modeled separately as distinct exposures in analyses. We fit separate negative binomial regression models relating the number of 311 requests to each neighborhood measure. We estimated unadjusted and adjusted incidence rate ratios (IRRs), along with 95% confidence intervals (CIs). A two-sided *p* < 0.05 was considered statistically significant. We presented results separately for all service requests and for those that mapped to an Ecological Model domain.

## 3 Results

### 3.1 Service requests and MAPS-A scores at PHDs

A total of 1,440 service requests were made across all PHDs during the study period. Of these, 1,204 (84%) corresponded to relevant domains of the Ecological Model. Table 3 shows the number of requests per PHD, the number of units per PHD, and PHD MAPS-A scores. Requests per PHD ranged from 28 to 247, and the number of units per PHD ranged from approximately 200 to 800. Overall total scores ranged from 13.6 to 24.9, with higher scores indicating more positive walkability.

**Table 3:** Frequency of all 311 service requests (01/31/2022–01/31/2023) and value of MAPS-A scores for 12 public housing developments.

Public housing development	1	2	3	4	5	6
Number of 311 incident reports						
0.1 miles radius	83	69	38	42	93	63
0.1 miles radius (requested reasons)^1^	61	51	32	28	87	56
Positive destinations and land use	3.7	7	5.7	2	4.3	4
Overall microscale positive	12.7	16.0	14.6	11.6	10.6	12.2
Overall total score	16.3	23	20.2	13.6	14.9	16.2
Overall microscale positive (active transport)	10.3	14	12.6	10.2	8.6	10.2
Overall total score (active transport)	14.0	21	18.2	12.2	12.9	14.2

Public housing development	7	8	9	10	11	12
Number of 311 incident reports						
0.1 miles radius	141	270	191	136	186	128
0.1 miles radius (requested reasons)^1^	111	247	148	122	150	111
Positive destinations and land use	3.3	8.7	7.3	10.3	6.7	6.5
Overall microscale positive	13.7	16.3	15.4	12.4	12.8	13.4
Overall total score	17	24.9	22.8	22.7	19.5	19.9
Overall microscale positive (active transport)	11.7	14.3	13.4	11.1	10.8	11.9
Overall total score (active transport)	15	22.9	20.8	21.4	17.5	18.4

1 Frequency of 311 service requests that correspond to the ecological model of four domains of active living.

Table 4 shows the unadjusted and adjusted models relating 311 service requests to each MAPS-A summary score. A one-unit increase in positive destinations and land use score was significantly associated with a 16% increase (unadjusted IRR [uIRR]: 1.16, 95% CI: 1.03–1.29) in the number of 311 requests. Adjusting for the number of units strengthened the association’s magnitude to 18% (adjusted IRR [aIRR]: 1.18, 95% CI: 1.03–1.33). We observed similar patterns in the models for the overall total score (uIRR: 1.10, 95% CI: 1.02–1.18; aIRR: 1.12, 95% CI: 1.03–1.22) and the overall total score for active transport (uIRR: 1.10, 95% CI: 1.02–1.18; aIRR: 1.12, 95% CI: 1.02–1.21). Therefore, higher neighborhood walkability scores were associated with a greater number of 311 service requests. The following subscales were not statistically significantly related to higher 311 request frequency: overall microscale positive (uIRR: 1.15, 95% CI: 0.96–1.34; aIRR: 1.14, 95% CI: 0.93–1.34) and overall microscale positive active transport (uIRR: 1.15, 95% CI: 0.95–1.35; aIRR: 1.15, 95% CI: 0.93–1.36). Limiting the adjusted analysis to 311 requests that mapped to an Ecological Model of Four Domains of Active Living physical activity domain did not change the patterns of statistical significance (Table 5).

**Table 4:** Unadjusted and adjusted associations between pedestrian environment features of neighborhood walkability (MAPS-A scale scores) and total 311 service requests.

MAPS-A scale scores	Unadjusted IRR (95% CI)^1^	Adjusted IRR (95% CI)^1^
	0.10 mile radius	
Positive destinations and land use	1.16** (1.03–1.29)	1.18* (1.03–1.33)
Overall microscale positive	1.15 (0.96–1.34)	1.14 (0.93–1.34)
Overall total score	1.10** (1.02–1.18)	1.12** (1.03–1.22)
Overall microscale positive (active transport)	1.15 (0.95–1.35)	1.15 (0.93–1.36)
Overall total score (active transport)	1.10* (1.02–1.18)	1.12* (1.02–1.21)

1 IRR, Incidence Rate Ratio; CI, Confidence Interval; All models adjusted for number of units.

Significance level: *** (<0.001), ** (<0.01), * (<0.05).

**Table 5:** Unadjusted and adjusted associations between pedestrian environment features of neighborhood walkability (MAPS-A scale scores) and total 311 service requests that correspond to the Ecological Model of Four Domains of Active Living.

MAPS-A scale scores	Unadjusted IRR (95% CI)^1^	Adjusted IRR (95% CI)^1^
	0.10 mile radius	
Positive destinations and land use	1.18** (1.05–1.32)	1.20** (1.04–1.36)
Overall microscale positive	1.15 (0.94–1.35)	1.13 (0.91–1.35)
Overall total score	1.11** (1.03–1.20)	1.13** (1.03–1.23)
Overall microscale positive (active transport)	1.15 (0.94–1.36)	1.13 (0.90–1.37)
Overall total score (active transport)	1.11* (1.02–1.19)	1.12* (1.02–1.23)

1 IRR, Incidence Rate Ratio; CI, Confidence Interval; All models adjusted for number of units.

Significance level: *** (<0.001), ** (<0.01), * (<0.05).

## 4 Discussion

In this exploratory cross-sectional study, we observed a positive association between neighborhood walkability and the frequency of 311 service requests at baseline in the 12 urban PHDs participating in Community Walks. Specifically, neighborhood walkability increased with increasing frequency of 311 requests.

There may be several factors driven by the broader neighborhood ecosystem underlying these results. Improved walkability may encourage more pedestrian traffic within these neighborhoods (28), which in turn may increase the likelihood that individuals navigating the environment will encounter and report issues via 311 service requests. This pattern may be influenced by gentrification occurring throughout Boston (29), as individuals from higher social positioning and privileged backgrounds move into these areas or commute through them, they may utilize the 311 system more frequently. Awareness of the 311 system may be greater in these demographics, driven by existing civic engagement, better information sharing, or structured organizing promoting municipal resources (30–32). Additionally, neighborhoods with greater walkability may also foster a stronger sense of community ownership and cohesion, motivating individuals to report concerns (33, 34). Lastly, municipalities may preferentially improve the built environment in neighborhoods that already generate more 311 requests, creating a feedback loop that reinforces these reporting patterns. While we cannot answer these questions in the current study, understanding these spatial reporting dynamics possibilities are targets for future research, particularly qualitative research to investigate these phenomena in-depth.

The immediate neighborhoods surrounding PHDs with low reporting via the ‘311’ system may warrant greater attention for potential investment in their built environments as a matter of equity given the likelihood of reporting environmental problems may vary by socioeconomic status (35). Using census-tract data from the city of Houston, Texas, Cook et al. found that 311 pothole reports were significantly less frequent in areas with lower average socioeconomic status and a higher percentage of Black and Hispanic residents, despite these areas having more potholes (36). Together, these studies suggest PHDs located in low-reporting areas may also benefit from proactive interventions to increase community cohesion, information sharing, and building awareness of existing resources to improve the built environment - elements associated with higher quality of life in communities (37). Increasing awareness of the 311 system could be one valuable intervention component, but given evidence that many 311 service requests remain unresolved, Data Team (38) other capacity-building components should be included in training efforts (e.g., how to connect with city counselors, with the transportation department, and with local divisions of neighborhood services). Recognizing these needs, in Community Walks (8), we partnered with the non-profit organization WalkMassachusetts (26) to provide in-person pedestrian advocacy training sessions for residents of Boston-area PHDs to share resources about how to advocate for better walking infrastructure in their communities. Effective pedestrian advocacy training has also been piloted among older adults, resulting in positive changes to the built environment (39). Such models demonstrate how organizations and communities can collaborate to promote healthier built environments.

This study has several limitations. Due to its cross-sectional design, we were unable to determine causality in the relationships between PHD walkability and the frequency of 311 service requests, or how those requests were handled (e.g., resolved, not resolved, ongoing). Therefore, we cannot directly evaluate if walkability improved because 311 requests were resolved. Examining this relationship would require longitudinal data of sufficient duration. Second, individual-level information on 311 service requests was unavailable, a limitation noted by others (40). For this reason, we could not confirm the 311 requests ascribed to a PHD were submitted by residents of that PHD. Instead, we assumed 311 service requests for problems arising within a 0.1-mile radius of the PHD’s address were generated by that PHD’s residents. Third, because PHDs varied in size, applying this rule may have captured service requests outside the PHD in some cases. Fourth, while the period of 311 data service requests overlaps with the walkability assessment time period, they are not completely aligned – which was necessary in order to capture one full year of 311 data (to account for changes in service request volume or type due to seasonality) prior to the start of Community Walks intervention activities. Potential unmeasured confounding is also a limitation, for example neighborhood civic engagement and resident turnover. Finally, these data are limited in their generalizability, given the limited sample size of 12 PHDs in our sample. Given these limitations, this study represents an exploratory starting point for future research, with a longitudinal study design, including information on service request resolution, in a wider geographical area, and the inclusion of qualitative data to explore residents’ perceptions of the 311 service request system in depth.

To date, 311 municipal datasets have been used in public health research (20–22, 30, 33, 36). In this study, we evaluated the relationship between 311 data about the built environment and a validated measure of neighborhood walkability at baseline in 12 Boston-based PHDs participating in Community Walks. We found a correlation between pedestrian environment features of neighborhood walkability and the frequency of 311 service requests. This study represents an exploratory starting point for future multi-level physical activity interventions in which 311 data could inform the selection of community-based activities to offer participants, the resources to advocate for, and targeted advocacy programming to improve neighborhood walkability, with the ultimate goal of increasing health-promoting physical activity behaviors.

## Data Availability

The raw data supporting the conclusions of this article will be made available by the authors, without undue reservation.
